# Microfluidic Platform for Cell Isolation and Manipulation Based on Cell Properties

**DOI:** 10.3390/mi8010015

**Published:** 2017-01-04

**Authors:** Caffiyar Mohamed Yousuff, Eric Tatt Wei Ho, Ismail Hussain K., Nor Hisham B. Hamid

**Affiliations:** Department of Electrical and Electronics Engineering, Universiti Teknologi PETRONAS, 32610 Tronoh, Malaysia; ismailhussain22@gmail.com

**Keywords:** microfluidics, cell isolation, cell manipulation, cell properties, lab on chip

## Abstract

In molecular and cellular biological research, cell isolation and sorting are required for accurate investigation of a specific cell types. By employing unique cell properties to distinguish between cell types, rapid and accurate sorting with high efficiency is possible. Though conventional methods can provide high efficiency sorting using the specific properties of cell, microfluidics systems pave the way to utilize multiple cell properties in a single pass. This improves the selectivity of target cells from multiple cell types with increased purity and recovery rate while maintaining higher throughput comparable to conventional systems. This review covers the breadth of microfluidic platforms for isolation of cellular subtypes based on their intrinsic (e.g., electrical, magnetic, and compressibility) and extrinsic properties (e.g., size, shape, morphology and surface markers). The review concludes by highlighting the advantages and limitations of the reviewed techniques which then suggests future research directions. Addressing these challenges will lead to improved purity, throughput, viability and recovery of cells and be an enabler for novel downstream analysis of cells.

## 1. Introduction

The human body has abundant cells of different types circulating in bodily fluids. Researchers are discovering a wealth of information about an individual’s state of health by analyzing the quantity, quality and type of cells present in these fluids. Selective isolation of cells by property or type from a heterogeneous mixture is the key enabler for a range of useful analysis, i.e., clinical diagnostics, monitoring, therapeutics or as a precursor to biomolecular analysis to understand complex functional interactions between tissues, organs and systems. For example, isolation of rare cells such as circulating tumor cells (CTCs) enriches cellular concentrations to an accurately measureable quantity thereby enhancing accuracy to stratify cancer patients or monitor treatment efficacy [1,2]. Isolation of circulating fetal cells contains genetic information of the fetus, which is useful to identify fetal autosomal abnormalities [3]. Isolation of sperm cells permits manipulations for in vitro fertilization [4] or forensic investigation [5]. Isolation of stem cells permits controlled differentiation of cells to repair or replace injured or diseased tissue [6] in stem cell-based therapies and research. Isolated red blood cells (RBC) and white blood cells (WBC, leukocytes) are required for haematological tests to diagnose a range of illnesses, including anaemia, infection, leukaemia, myeloma and lymphoma. Cancer biomarkers can be analyzed from clean plasma separated from blood for early cancer detection [7]. As isolation accuracy and throughput continues to improve, we anticipate that cellular level therapies will become feasible, i.e., the removal or rehabilitation of dysfunctional or diseased cells such as Human Immunodeficiency Virus (HIV)-infected T cells or malaria infected RBCs or the removal of pathogenic bacteria circulating in poisoned blood.

To move beyond proof of concept prototypes, these diverse applications of selective cell isolation require high accuracy and reproducibility [8]. Conventional cell isolation systems such as fluorescence-activated cell sorter (FACS), magnetic activated cell sorting (MACS) and centrifugation systems have demonstrated high robustness, accuracy and throughput and have high utility in industrial and lab settings. However, several limitations hinder deployment in more novel applications, such as the requirement for large sample volumes, high reagent consumptions, cross contamination of samples and expensive equipment cost. These systems achieve high throughput cell separation by labelling cells with surface markers or by separating cells based only on density property. To overcome these limitations, researchers devised various cell sorting technologies in miniaturized microfluidic platforms [9,10,11,12]. These new technologies enable selective isolation of cells using various other properties like size, shape, deformability, morphology, electrical properties, magnetic properties, compressibility, and also novel surface markers.

Thus far, there is no clear and superior technique to be universally adopted for selectively separating cells. This motivates our review of the different microfluidic mechanisms so that researchers could identify the most suitable technique for their application. We classify the microfluidic techniques by the cellular property employed to distinguish between cells. We believe this grouping is useful because we anticipate that future systems will utilize a suite of cellular features, in tandem or in succession, to achieve greater specificity and reproducibility of separation. Indeed, we discuss several recent innovations towards the paper that have demonstrated this concept. In each group, we highlight the advantages and disadvantages of each technique with respect to device performance and cell viability, throughput, cell recovery rate (yield) and output purity. Recovery rate or capture efficiency expresses the yield of target cells at the outlet compared to all target cells that entered the device. Cell purity expresses the sorting accuracy of sorting and is measured by the percentage of target cells over all cells present at the collection outlet. Cell viability at the most basic level are defined to those cells that are not dead. Throughput (flow rate) determines the number of processed cell per unit time.

## 2. Based on Induced Cell Properties by Labelled Antibody

### 2.1. Fluorescence-Activated Cell Sorting

The commercial FACS has become important for biomedical researchers and clinicians for purifying, sorting, counting and analyzing cells which cannot be easily cultured [13,14,15,16] such as stem cells, circulating tumor cells and rare bacterial species. The target cells are labelled with antibody-linked fluorescent dye. FACS systems separate target cells via a two-step process. An optical detection system first identifies the presence of fluorescent markers (which have been tagged to target cells only) in a stream of cells. Whenever a target cell is identified, the cell is electrostatically deflected to a collection reservoir by charging the individual cell encapsulated in a droplet. State-of-art FACS perform single-cell separations at rates of 50,000–100,000 cells/s and are capable of distinguishing between 14 and 17 different fluorescent markers [17,18]. There are many commercially available FACS systems which have been reviewed in detail by Picot et al. [19]. FACS systems require high capital investment, high reagent consumption, are only capable of binary separation, require lysing of red blood cells to enhance capture efficiency and may have cross contamination [20].

Microfluidic lab-on-chip devices circumvent these limitations through miniaturization. Instead of electrostatic charging and deflection, a variety of switching mechanisms employing different physics is used to manipulate and sort individual cells. Fu et al. [21] developed the first micro-fabricated FACS device using microfluidic valves for sorting and achieved a throughput of 20 cells/s. Grad et al. [22] designed an X-type junction device powered by two micro solenoid valves at the sheath inlets to separate labelled and unlabeled Fibroblasts at a throughput of 10 cells/min. Chen et al. [23] used a hydrodynamically gated valve. The target cells are separated by gating one outlet with negative pressure and achieved a throughput of 1.4 cells/s.

Sugino et al. [24] invented a novel switch which reversibly transitions from liquid to gel (solid) at elevated temperatures. A thermo-reversible gelation polymer (TGP) solution is mixed into the sample fluid containing cells. When a targeted cell is detected, the liquid mixture is heated to transition the TGP into a gel and deflect the target cell to a different outlet at a throughput of 2.8 cells/s. Target cells may be separated from other cells, but cannot be isolated from the sol-gel solution.

**Figure 1:**
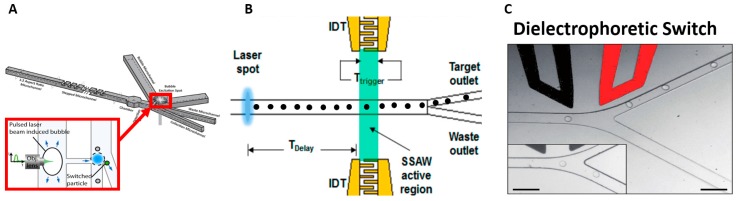
(**A**) Pulsed Laser beam induced bubble to push target cells, Reference [28], Reproduced with permission, Copyright Wiley-VCH Verlag GmbH & Co. KGaA; (**B**) Acoustic radiation force push the cell to target outlet, Reprinted with permission from Reference [29]. Copyright (2015) American Chemical Society; (**C**) Dielectrophoretic force attracts or repels the target cells, Reproduced from Reference [30] with permission of The Royal Society of Chemistry.

Perroud et al. [25] separated macrophages and Wang et al. [26] to sorted mammalian cells using an optical force based manipulation. A high-power infrared laser deflects target cells into a collection channel and achieved throughput of 20 cells/s. Chen et al. [27,28] used forces from an expanding vapor bubble to push targeted cells into a collection channel at throughput of 10,000 cells/s. The system creates an explosive laser bubble by tightly focusing a pulsed laser beam through a high NA objective lens to heat the liquid medium, as shown in Figure 1A. The system of Nawaz et al. [29], shown in Figure 1B, utilized acoustic force from 150 µs burst of acoustic wave to deflect targeted cells and achieved a throughput of 1200 events/s.

Several researchers have encapsulated cells within droplets so that dieletrophoretic force (see Section 6) may be used to push the target cells into the collection outlet [30,31,32,33]. The system of Baret et al. [30], shown in Figure 1C, sorted two different strains of *E. coli* and achieved throughput of 2000 cells/s. Target cells were encapsulated in a 12 pL emulsion droplet. Using a similar mechanism but with larger droplet size to improve cell viability, Mazutis et al. [31] demonstrated separation of antibody-secreting cells from non-secreting cells at a lower throughput of 200–400 cells/s.

Mechanically actuated microfluidic FACS systems have low throughput whereas systems actuated by other forces such as acoustic force, bubble expansion and dielectrophoretic force have 10–100× more throughput.

### 2.2. Magnetic Activated Cell Sorting (MACS)

Magnetic-activated cell sorting (MACS) is another antibody labelled approach similar to FACS. Cells of interest are tagged with marker-specific antibodies conjugated to magnetic labels. The fluid mixture containing tagged and untagged cells is flowed through a strong magnetic field. The magnetically tagged cells are directed into the collection channel by magnetic force. Many commercial extraction kits such as AutoMACS Pro separator (Miltenyibiotec, Bergisch Gladbach, Germany), CELLSEARCH (Janssen Diagnostics, LLC, Raritan, NJ, USA) are available on the market. These kits provide various antibody-labelled magnetic tags for isolation of leukocytes, circulating tumor cells, stem cells, viable cytokine secreting cells, to name a few. These commercial systems can isolate tagged cells with high throughput (10^9^–10^10^ cells/h), high purity and high recovery rate but require large samples and labels (magnetic particles), which is costly. Processing is done in batch mode and prolonged duration of operation increases the chance of cross contamination by non-specific binding with the magnetic particles. The review by Hejazian et al. [34] provides more insight into the fundamental physics and important design considerations for MACS systems.

Microfluidics-based magnetic activated cell sorting (µMACS) overcomes these limitations and provides a high purity and recovery rate while requiring fewer magnetic particles with continuous flow. To reduce the volume of magnetic particles needed for cell labeling, microfluidic devices create configurations that elicit stronger magnetic force by increasing the magnetic field gradients crossing the cells, either by increasing magnetic field strength or increasing proximity between magnetic source and tagged cells. However, there are limitations to the maximum allowable magnetic field gradients imposed by joule heating which reduces cell viability. Various configurations have been implemented using permanent magnets [35,36,37,38], electromagnets [39,40], and self-assembled magnets [41]. Osman et al. [42] designed a micromagnet array of Neodymium (NdFeB) films which act as permanent magnet with high magnetic field strength (10^6^ T/m).

**Figure 2:**
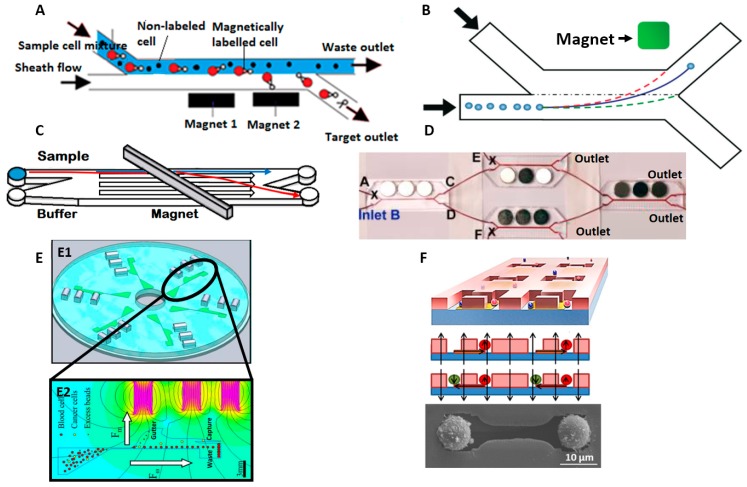
(**A**) A schematic of H filter for magnetic based separation (**B**) Viscoelastic focusing of magnetic particles Reproduced from Reference [48] with permission of The Royal Society of Chemistry; (**C**) Angled permanent magnet configuration Reproduced from Reference [49] with permission of The Royal Society of Chemistry; (**D**) Cascade magnetic separation stages, Adapted with permission from Reference [50]. Copyright (2014) American Chemical Society; (**E**) Schematic of Lab on disc chip with microfluidic channels, visible in green, and magnets as silver (E1) and Inset view of one of the channel in E1, where simulation shows, blood cells, excess beads collected at waste, target cells at capture and bead waste at gutter (E2), Reproduced with permission, Reference [52], Copyright Wiley-VCH Verlag GmbH & Co. KGaA; (**F**) Patterned micromagnets over microwells to isolate magnetically labelled cells, Reproduced from Reference [54], with the permission of AIP Publishing.

Many µMACS methods used the H channel structure to separate target cells from a mixture with two inlets and two outlets [43,44,45,46,47], shown in Figure 2A. The mixture of magnetically labeled and non-labeled cells are introduced into one of the inlets and sheath flow is introduced into the other inlet at the same flow rate. Laminar flow in the micro channel keeps the streams distinct and permanent magnets placed beside the streams attract magnetically tagged cells to cross the stream into the collection channel. By optimizing the placement and distribution of magnetic force, Del Giudice et al. [48] achieved up to 96% separation efficiency at flow rate of up to 4 µL/min, using the concept illustrated in Figure 2B. Cells from multiple target groups can be tagged with differently sized magnetic particles and experience different magnetic force and deviations into different outlets [35].

Among the efforts to improve the performance of magnetophoresis system, Forbes et al. [49] developed a comprehensive numerical model as a tool to design magnetophoretic systems. With aid from the tool, they demonstrated separation of magnetically labeled breast adenocarcinoma (MCF-7) cells using lateral magnetophoresis with an angled permanent magnet configuration as shown in Figure 2C. Lee et al. [50] isolated magnetically tagged *E. coli* bacteria from whole blood and improved efficiency to almost 100% at throughput of 60 mL/h by cascading three similar separation stages in series and parallel as shown in Figure 2D.

Kirby et al. [51] combined centrifugation with magnetophoresis to isolate magnetically labelled cells in a device depicted in Figure 2E. Centrifugation forces push cells radially outward along the microchannels as the device spins at high speeds. Magnets close to the channel pull the target cells into a collection chamber while the remaining unlabeled cells are collected at the end of the microchannel. They successfully demonstrated the isolation of MCF-7 cancer cells from whole blood and HIV/AIDS relevant epitopes from the whole blood [52] with high sensitivity of 1 cell per mL at a capture rate of 88% and 92%, respectively.

As an alternative to focusing, Hoshino et al. [53] demonstrated capture of magnetically tagged cancer cells from a continuous flow of blood using a microfluidic device with magnets arrayed at the bottom of the channel. They achieved a sensitivity of 1000 cells down to 5 cells per mL with a capture efficiency of ~86% for two cancer cell lines at a throughput of 10 mL/h. Similarly, Shields et al. [54] created a device with patterned micromagnets over microwells to isolate magnetically labeled CD4+ lymphocytes from blood with 95% accuracy, shown in Figure 2F.

Magnetic-label sorting compares favorably against fluorescent-label sorting because identification and actuation of target cells are performed at the same step. However, the throughput reported in the literature is low (4 μL/min–60 mL/h) to allow sufficient duration for the magnetic force to displace tagged cells into the collection channel. Also, antibodies and the attached magnetic labels are difficult to remove from the surface and can alter the properties of target cells. It remains an open challenge to detach magnetic beads from labelled cells after separation [17].

## 3. Separation Based on Cell Surface Markers Properties

Panning is a classical method to isolate cells where a surface is functionalized with antibodies and target cells flowing through the surface are captured and tightly bound by antibody coupling. This technique is frequently used but limited by low capture efficiency and purity levels. To compensate, large number of cells (10^5^–10^7^) are required which makes panning unsuitable for applications with a small volume of analytes such as small cell populations harvested from organs and tissues or applications with high false positive rates from non-specific bindings [55].

To improve yield purity and capture rate over classical antibody treated surfaces, microfluidic implementations have various innovations in the design of surfaces [56,57,58,59,60,61,62,63,64,65]. Conventionally, CTC cells are selectively captured on surfaces functionalized with antigen Epithelial cell adhesion molecule (anti-EpCAM) antibodies [56]. Although expressed in most CTCs, the surface antigen EpCAM is notably absent on some non-epithelial and melanoma cells. Capture efficiency also decreases as flow rate is increased. Gaskill et al. [59] increased capture efficiency up to 98% at a flow rate of 18.7 µL/min by adding a second adhesion molecule, E-selectin, to the microchannel surface. Through rapid bonding and bond-breaking of leukocytes to E-selectin, leukocytes accumulate near the capture surface and impede CTC flow to maximize CTC cell contact with anti-EpCAM on the capture surface.

Kurkuri et al. [60] achieved capture efficiency of cancer cells between 80% and 90% at throughput of 48 µL/min by increasing the area of the capture surface. They created microposts on the polydimethylsiloxane (PDMS) capture surface and functionalized with EpCAM. Mitchell et al. [61] developed an alternative design to increase surface area. They immobilized nanostructured Halloysite nanotubes (HNT)-coated with E-selectin on the capture surface to increase the surface area to capture CTC from leukocytes. They achieved 90% purity at a throughput of 0.04 mL/min.

Captured cells can be released with aptamer functionalized surfaces. Zhu et al. [62] demonstrated reversible capture and cell viability post-release on T-Lymphoblast human cell lines (CCRF-CEM) and Zhang et al. [63] demonstrated selective capture of *E. coli* from a mixture with different bacterial species. Target cells are captured via affinity binding and non-target cells are removed by washing. When the surface temperature is increased (48 °C for 2 min) by micro-heaters arrayed on the functionalized surface, aptamer binding is disrupted and the target cells are released. They were able to capture the target cells of approx. 250 cells/mm^2^. Jeon [64] also developed a conductive nano surface to electrically release and retrieve captured CTCs. The capture surface was first electrodeposited with biotin-doped conductive polypyrrole and subsequently functionalized with biotinylated antibodies. Up to six different cell lines of CTCs can be selectively captured by adhesions through the various antibody types. Captured CTCs are successfully released through application of a voltage pattern to the conductive polypyrrole, which liberates the biotin-CTC conjugate for collection. With this method, they achieved capture and release efficiency of 97% and 95%, respectively, at a flow rate of 1.2 mL/h.

Instead of selectively immobilizing target cells, Bussonie et al. [65] demonstrate an alternative principle of separating cell types according to differences in adhesion strength to the surface. They captured HEK293 and A7R5 cells on a surface coated with LiNbO_3_ and using a surface acoustic wave to detach the adherent cells, they achieved sorting purity of 97% and sorting efficiency of 95%.

Separation by surface marker properties has been popularly applied to isolating CTCs from a heterogeneous mixture of whole blood. However, it remains challenging to improve the selectivity for other cell types because many markers expressed on the cell surface are not unique to a particular cell type.

## 4. Separation Based on Size, Shape, and Deformability

Cell separation by physical properties such as size, shape, and deformability are promising avenues for real-time diagnostic and clinical applications. This label-free approach does not require expensive chemical reagents or antibody labelling thus reducing sample preparation time while improving throughput and cell viability. The various techniques that utilize these physical properties to isolate cells are discussed below.

### 4.1. Membrane Filtration (Size)

**Figure 3:**
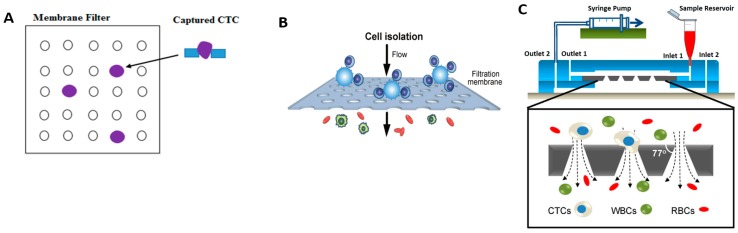
(**A**) A schematic of membrane filtration; (**B**) polydimethylsiloxane (PDMS) membrane filter, Reference [78] Reproduced with permission, Copyright Wiley-VCH Verlag GmbH & Co. KGaA; (**C**) Conical Membrane Filter, Adapted by permission from Macmillan Publishers Ltd.: [Nat. Commun.], Reference [80], copyright (2014).

Membrane filtration in microfluidic devices utilize thin membrane layers with micro-pores of predefined size, geometry and spatial distribution. Target cells, predefined by size, are not permitted to flow through the membrane whereas other cell types and the liquid may pass freely. The membrane filter is made from various polymer material like thermoplastic material [66], parelyene material [67], photo definable material [68], and PDMS membrane [69]. A schematic depicting membrane filtration is shown in Figure 3A. Cell separation by membrane filtration has been demonstrated on numerous cell types; for example, Fatanat et al. [66] isolated rare oligodendrocyte progenitor cells (OPCs) from rat brain tissue, Zheng [67] isolated CTC from whole blood and Adams et al. [68] isolated MCF-7 cell lines from whole blood.

Commercially available CTC separation systems like Parsortix system (ANGLE plc., Guildford, UK) use size-based filtration and achieve a capture rate of 66% and a release rate of 61% at throughput of 0.17 mL/min [70] and fewer depleted leukocytes (200–1000 cells) [71,72]. It outperforms commercial labelled sorting systems like ScreenCell (ScreenCell, Sarcelles, France) [73], IsoFlux (Fluxion Biosciences, Inc., San Francisco, CA, USA) [74], CellSearch system, and AutoMACS. IsolFlux has a capture rate of 40% whereas CellSearch has a release rate of 19% [70].

Although conceptually simple, careful design of membrane filters is crucial for good performance. To maximize throughput, accuracy and precision of cell separation, membranes must have good uniformity of pore geometry, high porosity [67,75,76,77] and be thermally and mechanically stable. Proper choice of through-hole diameter is the crucial design parameter to separate the target cells. The membrane fabrication process must give uniform microstructures and researchers have endeavored to reduce fabrication complexity [69,78] to improve quality. Membranes are also optically transparent to facilitate further downstream analysis. Clogging of membrane pores by large particles is a frequent problem which rapidly degrades capture efficiency. If cross-flow injectors are added, clogging can be minimized because non-adherent cells are flushed away from the membrane surface.

Chen et al. [78] demonstrated an approach to control the size of filtrates artificially and separate leukocytes from whole blood. In this system, shown in Figure 3B, leukocytes were conjugated to microbeads and were readily trapped on the PDMS microfiltration membrane (PMM) whereas other non-target cells could pass through the membrane freely. The diameter of through holes in PMM was smaller than the microbeads but greater than unbound leukocytes. The device achieved throughput of 20 mL/min with 97% capture purity. Furthermore, the leukocytes trapped on the PMM could be analyzed with immunophenotyping assays like AlphaLISA [79], i.e., to determine cytokine concentrations. Compared to conventional whole blood stimulation assays, like ELISA (enzyme-linked immunosorbent assay) or ELISpot (Enzyme-Linked ImmunoSpot), this new process yielded a 10-fold reduction in processing time.

Similar work also reported recently by Fan et al. [69] for isolation of CTC with a capture rate of >90% and relatively higher processing throughput at 10 mL/h. This method is found to be the best choice for CTC separation and downstream analysis. Other than uniform circular pores, conical pores designed by Tang et al. [80], as shown in Figure 3C, with cross flow components has shown promising results in capturing CTCs, with a high efficiency of 96% and a high purity at flow rate of 0.2 mL/min. In addition to this, captured CTCs can be cultured in the same device for further analyses and to clearly understand the cell behavior, which is generally adopted.

Membrane filtration has disadvantages, i.e., cell proliferation and release from the filter pores are restricted [81]. Also, cells are often washed with fixatives to prevent lysis during filtration [66,67,68]. Zhou et al. [82] attempted to overcome these limitations by designing a separable bilayer (SB) microfilter to separate viable CTC cells from blood with efficiency of 83% and cell viability of 74% at 0.4 mL/min flow rate. The gap between the two layers and the pore alignment are designed to induce less mechanical stress on CTCs, thus improving cell viability for further analysis downstream.

### 4.2. Inertial Separation (Size)

Inertial separation utilizes inertial forces within flowing fluid to deflect cells. By careful design of microchannel dimensions and geometry, cells of different sizes will migrate to different positions along the micro-channel induced by various forces, i.e., smaller cells will be dominantly affected by dean forces which are generated by a lateral, secondary-vortex flow along a spiral channel whereas the larger cells will migrate under inertial lift forces. Inertial lift forces arise from two competing forces, namely the shear induced lift, which pushes cells towards the channel wall due to the interaction with the fluid velocity profile, and the wall induced lift, which pushes cells away from the channel wall due to interactions at close proximity to the channel wall. To generate sufficient displacement force, the cell–fluid mixture must flow at a sufficiently high velocity with Reynolds number, Re >> 1 [83]. Di Carlo first showed the utility of inertial forces by demonstrating cell focusing and cell deflection in a variety of different channel geometries such as asymmetrically curved channels [84], straight high aspect ratio channels and contraction-expansion arrays (CEA) [85].

**Figure 4:**
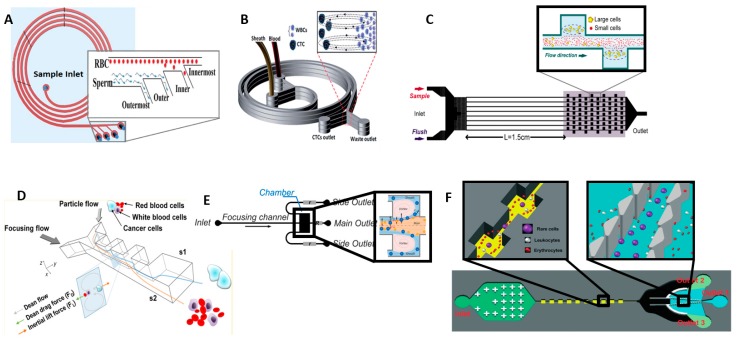
(**A**) Spiral microchannel for sperm cell and RBC isolation, Reproduced from Reference [87] with permission of The Royal Society of Chemistry; (**B**) Stacked spiral channel for high throughput applications, Reproduced from Reference [102] with permission of The Royal Society of Chemistry; (**C**) Contraction-Expansion with Vortex Aided Separation, to trap larger cells in expanded reservoir region due to the difference in the lift forces on the cells Reproduced from Reference [85], with the permission of AIP Publishing; (**D**) Contraction-Expansion array separation, Reprinted with permission from Reference [104]. Copyright (2013) American Chemical Society; (**E**) Vortex-aided inertial microfluidic with siphoning outlet, Reproduced from Reference [105], with the permission of AIP Publishing; (**F**) Inertial force and Steric hindrance at the outlet for effective separation, Reproduced from Reference [106] with permission of The Royal Society of Chemistry.

Gregoratto [86] demonstrated separation using spirally shaped microchannels at a flow rate of 2 mL/min. Son [87] isolated non-spherical cells such as sperm cells from RBCs and successfully recovered 81% of non-motile sperm at two outer wall outlets and 99% of RBCs are recovered at two inner wall outlets at a flow rate of 0.52 mL/min with the system shown in Figure 4A. Other groups have demonstrated successful sorting using variations of the spiral configuration for blood plasma, CTCs and malaria-infected cells [88,89,90,91]. Jimenez et al. [92] demonstrated separation of viable waterborne pathogens like Cryptosporidium parvum. Hong et al. [93] utilized two 90° curved channels to separate viruses, bacteria and larger aerosols into three outlets, thus showing an improvement over traditional bioaerosol sampling methods.

Following this, Papautsky and Bhagat invented several spiral architectures for continuous multi-particle separation [83,94,95,96,97,98] operating at higher flow rates of up to 3 mL/min. They invented a two-stage separation technique to separate a range of different cell types including blood and tumor cell. The first stage utilizes inertial lift force in low aspect ratio channel to separate cells into two equilibrium streams near the top and bottom walls of the microchannel. Streams from the first stage enter the second stage where rotationally induced lift forces filter cells with enhanced efficiency. The achieved 99% efficiency separating Human Prostate Epithelial (HPET) tumor cell from diluted blood [97]. As shown in Figure 4B, Warkiani invented a multiplexed system with three stacked spiral channels and achieved complete isolation of various blood components like plasma [99], CTCs [99,100,101,102], Malaria infected cells [100] and CHO (Chinese Hamster Ovary) cells [99] with this design, achieving a high throughput of 7.5 mL/5 min [88].

Instead of curved geometries, contraction-expansion channels, shown in Figure 4C, can also be utilized for separation as demonstrated by Hur et al. [85]. At high flow rate, microscale vortices develop in the expansion region of the channel to entrap the larger sized cancer cells while smaller sized blood cells continue unhindered in a focused flow. Captured cells are later released by reducing the flow rate through the channel. Cells can be processed at a throughput of upto 7.5 × 10^6^ cells/s at flow rate of 4.5 mL/min. The same group developed a high throughput vortex chip (Vortex HT) [103], by adding two parallel channels and 1.5× more serial reservoirs for cell capture which improved the capture efficiency from 20% to 83%. This design achieves high throughput of 8 mL/min and purity of 85% for separation of MCF-7 breast cancer cells from whole blood.

Lee et al. [104] achieved a high recovery rate of 99.1% and throughput of 1.1 × 10^8^ cells/min using a two-stage CEA channel, shown in Figure 4D. The design utilized a sequence of high-aspect-ratio channels with alternating large and small widths which causes blood cells to focus close to the channel sidewalls due to inertial lift forces. By selecting the width of the pinching or narrow regions to be on the order of the largest cell size (cancer cells), CTCs are refocused along the channel axis while the rest of the cells remain aligned around the sidewalls. A bifurcating outlet at the final stage funnels the segregated CTCs, red blood cells and peripheral blood leukocytes to different outlet channels. Using only a single CEA channel, Wang et al. [105] created siphoning outlets beside the entrapment chambers, shown in Figure 4E, to continuously draw off 86% of the large cells trapped in the vortices and achieved 99% purity when separating RBCs from large polystyrene particles at a flow rate of 0.5 mL/min. Shen et al. [106] also designed a multistage device by combining inertial microfluidics with a size-based pre filtering with CEA and post filtering with steric hindrances to sort blood cells and tumor cells MCF-7 and HeLa cells, shown in Figure 4F. This combined stage provides >90% recovery rate at throughput of 2.24 × 10^7^ cells/min with >92% viable cells were found.

Zhou et al. [107] studied the design parameters responsible for effective trapping and separation in symmetric, rectangular expansion channel, i.e., dimensions of expansion region, sample concentration, threshold Reynolds number designed for trapping particles at higher flow rate of *Re* = 180, and achieved 98% trapping efficiency.

High throughput and fine cutoff between sizes can be achieved but the challenge remains. Flows populated densely with cells will destabilize the separation efficiency and this places upper limit on maximum achievable throughput. Also, size alone may not be a sufficiently discriminative feature between cell types.

### 4.3. Deterministic Lateral Displacement (Size, Shape, and Deformability)

**Figure 5:**
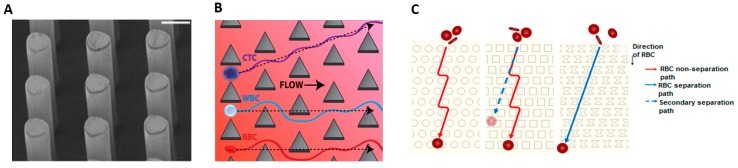
(**A**) Circular Pillar array for white blood cells (WBC) and red blood cells (RBC) separation, Adapted by permission from Macmillan Publishers Ltd.: [Nat. Commun.], Reference [110], copyright (2014); (**B**) Triangular Pillar array for Circulating Tumor Cell (CTC) Separation, Reproduced from Reference [113], with the permission of AIP Publishing; (**C**) Circular, Square, and I-Shaped pillars for RBCs separation, Adapted by permission from Macmillan Publishers Ltd.: [Nat. Commun.], Reference [114], copyright (2013).

The deterministic lateral displacement (DLD) approach separates cells based on differences between the combination of size, shape and deformability. Cells are flowed through an array of micro-structures or micropillars, as shown in Figure 5A, and are differentially displaced when the flow forces cells around the obstructing micro-structures. By carefully designing the positional offset of successive rows of micropillars, cells larger than a designed critical radius will be gradually deviated from the initial flow whereas small cells will flow along the streamline of the initial flow [108].

Researchers investigated the efficacy of different pillar shapes. Circular pillars were popularly studied for separation of various cell types [108,109,110,111]. For instance, Holm et al. [108,109] successfully isolated RBCs from diluted blood. Liu et al. [112] subsequently demonstrated that triangular pillars are better suited for cell types with large deformations because cells deform minimally around the pillar apex. They achieved separation efficiency between 80% and 99% for separation of various cancer cell types (MCF-7 and MDAMB231) from blood at a throughput of 2 mL/min. Loutherback et al. [113] achieved a higher throughput of 10 mL/min by a novel arrangement of triangular pillars shown in Figure 5B, which isolates CTCs from blood with capture efficiency greater than 85% and no impediments to cell viability. Zeming [114,115] studied the efficacy of various micropillar shapes i.e., circular, square, I-shaped, T-shaped, L-shaped and anvil, as shown in Figure 5C. The I-shaped pillar was most effective at separating non-spherical from spherical cells. The protrusion and groove structure induces rotational motion on the non-spherical cells thus increasing its effective diameter. The net effect of the I-shaped pillar is to enhance the disparity of sizes between the spherical and non-spherical cells. Another recent innovation is to reduce the downstream gap size between micropillars [116]. This optimization does not restrict throughput but enhances the separation efficiency of RBC up to 100% at a flow rate of 0.2 µL/min from whole blood.

DLD devices suffer from several key limitations. Individual pillars are prone to defects in size, shape and height [117] and any structural irregularities within the array tends to blur the streams, degrade separability of cells and increase the possibly of stiction and blockages. Long channel lengths are required to achieve significant lateral displacement [118].

## 5. Separation Based on Size, Density and Compressibility

### 5.1. Centrifugation and Pinched Flow Fractionation (Size and Density)

Fluids with a mixture of densities have the natural tendency to sediment into layers of increasing density under the influence of gravitational forces. Centrifugation accelerates this separation process by means of high magnitude centrifugal force generated from high speed rotation. Low et al. [119] recently reviewed commercial systems that employ centrifugation for CTC isolation. Commercial centrifugation systems are designed with powerful motors to circulate large volumes of liquid.

**Figure 6:**
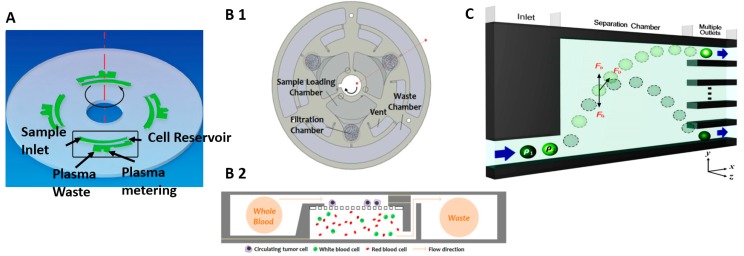
(**A**) The disc containing four identical plasma separation structure, Reproduced with permission from Reference [123], ^©^ IOP Publishing. All rights reserved; (**B**) The top view of the CTC-isolation disc showing detailed microfluidic features (B1) and the schematic illustration showing the working principle of the CTC-isolation disc (B2), Reprinted with permission from Reference [124]. Copyright (2013) American Chemical Society; (**C**) Conceptual microfluidic design for density based separation at different heights. Reprinted from Reference [128], with the permission of AIP Publishing.

In contrast, microfluidic implementations of centrifugation are designed to process small volume of fluids between 5 and 2000 µL [120,121]. Several researchers have adapted the Compact Disc (CD) systems to achieve high speed rotational motion with low cost commodity parts. The microfluidic channels are created on the CD and rotated at very precise and consistent speeds on the audio playback system. Li [122] achieved 92% separation efficiency of blood plasma from whole blood at 2000 rpm. Burger [123] also demonstrated up to 80% of plasma extraction within a short, 2 min interval with the system shown in Figure 6A. Lee et al. [124] used centrifugation to push cells in unprocessed whole blood through a membrane filter, depicted in Figure 6B, and successfully isolated CTC with capture efficiency of 61%.

In pinched flow fractionation (PFF), cells are focused in a narrow channel and abruptly enter an expansion area and come under the influence of gravity, drag and buoyancy forces. Cells that are less dense than the surrounding medium will be lifted to the upper regions of the expanded chamber. Cells with higher density will experience greater gravity force and flow towards the bottom of the chamber. This technique was demonstrated for isolation of spherical and non-spherical particle having similar density [125], particles of different size [126], CTC from WBC [127]. Song et al. [128] observed that the height differences between two cells of different densities could be amplified by decreasing the flow rate, depicted in Figure 6C. Morijiri et al. [129] also demonstrated PFF separation but combined with centrifugation to generate a larger and controllable sedimentation force to enhance the separation between particles of different densities at a throughput of 2060 µL/h.

Many cell types have similar density and size and centrifugation is not able to distinctly separate between them. Though some literature reported the high throughput separation using a centrifugal based approach, still this method is not sufficiently demonstrated for different cell types including rare cells, white blood cells, and whole blood cell mixture.

### 5.2. Acoustophoresis (Density and Compressibility)

**Figure 7:**
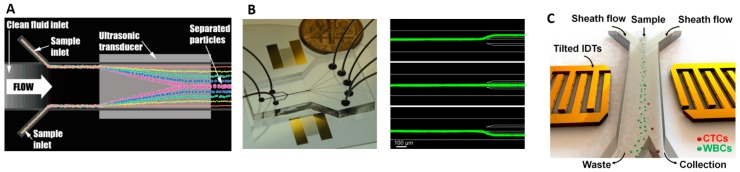
(**A**) Illustration of a suspended particle crossing transducer is pushed to center channel based on their acoustic properties, Adapted with permission from Reference [131]. Copyright (2007) American Chemical Society; (**B**) standing surface acoustic wave (SSAW)-based cell-sorting device with three inlets and five outlets and 15 µm fluorescent particle are directed to three different outlet channels by adjusting the applied frequency (14.5 MHz, SAW off, and 13.9 MHz), Reproduced from Reference [135] with permission of The Royal Society of Chemistry; (**C**) Schematic illustration of a high-throughput the angle of the standing surface acoustic waves (taSSAW) device for cancer cell separation, Reproduced with permission from Reference [130], Copyright (2015) National Academy of Sciences.

Acoustophoresis based cell isolation is a technique which uses the density and compressibility properties of a cell to distinguish different cell types. For example, CTCs and WBCs have different compressibility [130]. In acoustophoretic microdevices, high-intensity sound waves [131] interact with the microchannel to generate pressure gradients that push cells into specific spatial locations. The acoustic waves reflect off the microchannel walls to establish a standing wave pattern within the microchannel. Cells flowing through the standing wave are moved towards high pressure or anti-pressure node by radiation forces. The magnitude of the radiation force depends on the volume, density and compressibility of the cell, the surrounding medium and the amplitude and wavelength of acoustic wave. Cells with greater density and compressibility than the surrounding fluid will migrate towards the pressure node. Bands of cells, grouped by density and compressibility, form across the microchannel. Since flow is laminar, cells will hold the position in the band even after crossing the acoustic zone and conveniently collected at separate outlets [132], shown in Figure 7A.

Yang et al. [133] demonstrated separation of apoptotic cells from viable cells of the same type. They achieved 94% recovery of viable cells with 91% purity at throughput of 5 mL/h. Burguillos et al. [134] studied the impact of acoustophoretic separation on cell viability, proliferation and cell response to subtle phenotypic changes. They demonstrated that acoustophoretic processing did not affect cell viability of prostate cancer cells nor the respiratory functions for human thrombocytes and leukocytes.

Ding et al. [135] showed that particles could be displaced towards different outlets by modulating the frequency of a standing surface acoustic wave (SSAW) using the prototype shown in Figure 7B. To improve separation efficiency and increase separation distance between cell types, they tilted the angle [136] of the standing surface acoustic waves (taSSAW) relative to the channel as shown in Figure 7C to generate multiple pressure nodal lines. This configuration increases the likelihood of capturing cells within one of the nodal lines and they achieved a recovery rate of 71% with a purity of 84% when separating cancer cells from blood. By optimizing inclination angle, channel dimension and SSAW input power, they improved the cancer cell recovery rate to 83% at throughput of 20 µL/min [130]. Cell types are separated by displacements between 100 and 250 µm which is significantly larger than the displacements achieved by inertial forces and dielectrophoresis (DEP) [137,138].

Acoustic-based separation has a good recovery rate and high purity but lacks the limitations of operating at low flow rates as discussed in the literature so far. Secondly, even though acoustic based separation was applied to sort CTCs from other cells like WBCs, still compressibility properties of clinical CTCs and other rare cells are largely undocumented and unknown. Therefore, it initiates future studies to establish ideal physical property profiles from different cell populations to optimize the device performance.

## 6. Separation Based on Electrical Properties

Dielectrophoresis (DEP) is based on the principle that a force is induced on a dielectric particle when it is placed in a non-uniform electric field. Cells are not charged but polarized in the non-uniform field. Polarized cells experience a translational force, called DEP force, which either deflects (negative DEP) or attracts (positive DEP) towards the region of maximum field strength. The polarization acquired by cells depend on the cellular conductivity and permittivity, inherent polarizability of the fluid and also the magnitude and frequency of the applied electric field. To enhance the electric field gradient and the force induced on the cells, liquid solutions with free electrolytes are frequently added to the cell mixture. DEP was applied to sort CTC [139,140,141], blood cells [142,143,144], immune cells [145,146] and pathogens [147,148].

**Figure 8:**
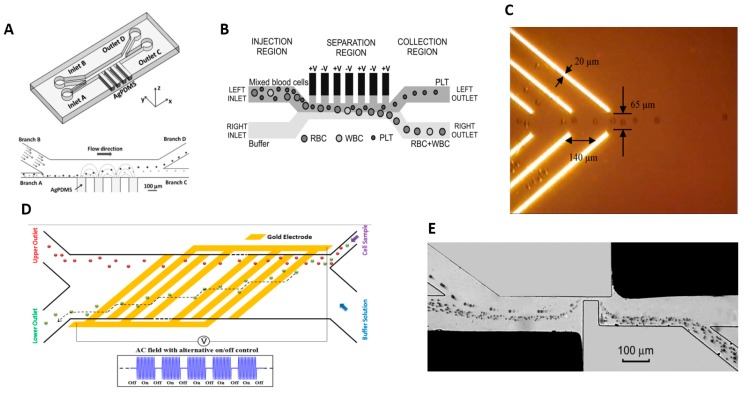
(**A**) PDMS microdevice with 3D sidewall composite electrodes with separation mechanism in the fabricated microdevice, Reference [137], Reproduced with permission, Copyright Wiley-VCH Verlag GmbH & Co. KGaA; (**B**) Liquid Electrodes placed at the left side of the channel for dielectrophoresis (DEP)-based platelets, RBCs and WBCs separation, Reproduced from Reference [142], with the permission of AIP Publishing); (**C**) Cell Focusing using Virtual electrodes (ODEP) Reprint with permission, from Reference [149], Copyright Elsevier (2008); (**D**) An oblique interdigitated electrodes for the continuous flow DEP based microfluidic cell separation, Adapted from Reference [144] with permission of The Royal Society of Chemistry; (**E**) Copper electrode with insulating PDMS hurdles for 5 µm, 10 µm and yeast cell separation Reproduced from Reference [138] with permission, Copyright Elsevier.

A common DEP strategy is to induce deflections of target cells to separate them from a flow. The basic design comprises of a H channel with sample and sheath flow inlets, two outlets and sidewall electrodes that generate the electric field gradient across the microchannel [136,140,144,146]. Thick metal electrodes are needed to generate uniform electric field gradients over the entire height of the microchannel [144,149] but are challenging to fabricate by metal deposition. To overcome these limitations, Lewpiriyawong et al. [137] developed three dimensional Ag-PDMS composite electrodes in the sidewall of the microchannel, shown in Figure 8A. Piacentini et al. [142] demonstrated a design with liquid electrodes, shown in Figure 8B, and successfully isolated RBCs and platelets with 98% purity and recovery rate. To enhance separation distance without undue increases in the electric field amplitude. Researchers also demonstrated optically induced DEP (ODEP) on a photoconductive sheet of amorphous silicon. The ODEP or virtual electrode was created on the sheet by projecting a pattern of light from a commercial projector as shown in Figure 8C. Different electrode geometries could be created in ad hoc fashion by simply changing the pattern of projected light. Researchers have achieved separation of prostate cancer cells (PC3) from leukocytes and viable sperm cells from non-motile cells [150,151,152] with separation efficiency of >85% at a particle velocity of 150 µm/s.

Song [144] used an array of oblique interdigitated electrodes to separate hMSC cells from osteoblasts and achieved separation efficiency of 92% at throughput of 5.4 µL/min with purity of 97%. The osteoblasts where polarized and deflected by DEP force (positive or negative) following a zig-zag trajectory, shown in Figure 8D. Ling et al. [152] invented a novel method of deflecting target cells from a flow using an array of triangular-shaped electrodes at the base of the microchannel. Due to its geometry, the apex of each electrode has a strong electric field, which attracts target cells by positive DEP. Target cells are incrementally deviated from the main flow by each peak it traverses while the untargeted cells remain undisturbed in the flow. They separated live fibroblast cells (NIH-3T3) from dead cells in Swiss mice, and osteosarcoma cells (MG-63) from erythrocytes with 82.8% separation efficiency at a throughput of 1302 cells/min.

Instead of deflecting cells in a continuous flow, the system designed by Jen et al. [153,154] required the cell fluid mixture to be dropped onto patterned planar electrodes. The electrodes form a concentric circular pattern. By activating and deactivating the electric potential between adjacent electrode pairs, the target cells are gradually moved to the center of the planar electrode by positive DEP force. They separated HeLa cells from MCF-5 cells with separation efficiency above 80%.

An alternative DEP strategy is to trap target cells by DEP forces and later release them for collection. Many researchers opted for array arrangements and novel electrode geometries surrounding the microchannel. Microwell arrays attract and trap target cells inside the wells by DEP forces. The target cells may be subsequently released into a separate collection reservoir by removing the voltage applied to the microwells. The design is similar to microliter well plates but scaled down in size and fabricated by drilling holes in a three-dimensional laminate made from stacked layers of graphene [155] or copper [156] sheet (interleaved with insulating layers). The resulting microwell walls act as electrodes. Another common design using extruded electrodes generate strong DEP forces, which improves throughput and affords better trapping efficiency up to a flow rate of 12 µL/min. Targeted cells such as HL-60 cell [157], polystyrene particles, drosophila cells [158], and viable yeast cells [155,159] are trapped by the electrodes in the microchannel with positive DEP while non-target cells are flushed to the outlet via negative DEP. Arrays of extruded electrode posts have been fabricated from gold [157], carbon [158] and highly doped silicon [160].

New electrode configurations are being invented and some have not yet been tested on live cells. Yafouz et al. [161] demonstrated a novel electrode configuration combining positive and negative DEP to trap polystyrene particles from 1 to 15 μm at separate locations around the electrode. Using a ring-shaped microarray of dot electrodes, the larger size particles were repelled and concentrated in the center of the ring shape by negative DEP while the smaller sized particles were attracted to the edge of the dot by positive DEP. Lapizco et al. [162,163] trapped particles between an array of insulating posts within a microchannel. When a direct current (DC) potential is applied between the inlet and outlet of the microchannel, the insulating posts divert electric field lines through the spaces between the posts, thereby increasing the local electric field strength there. As a result, a strong positive DEP force attracts and traps target cells between the posts.

To generate sufficient forces for deflection or trapping, high electric fields are needed for DEP but it inflicts membrane stress and induces joule heating on cells, which may eventually lead to cell death. Devices with insulating hurdles, as shown in Figure 8E [138], were designed to minimize the deleterious effects of high electric field exposure to cells. The hurdle is a constricted region joining both sides of the channel. The constriction compresses the electric field lines thus creating a localized region of high field strength. Cells experience high electric fields and deflection by DEP forces only within this region, thus minimizing exposure to its dangerous influence. Variations on the hurdle shape have been proposed, i.e., multiple rectangular insulating hurdles [164], triangular hurdles within an H-shaped microchannel [165,166] and S-shaped hurdles [167]. Hurdle designs have been used to separate biological cells [168] and DNA molecules [169].

However, DEP remains challenging to deploy in a reliable manner because dielectrophoretic forces are highly sensitive to buffer conditions (e.g., salt concentration) and achieve low throughput (typically <100 cells/s per microchannel) [133]. DEP based systems have thus far not demonstrated separation of multiple target cells. Several review articles have recently enumerated the advantages of DEP techniques [170,171].

## 7. Separation Based on Intrinsic Magnetic Properties of Cells

Unlike Section 2.2 whereby magnetic properties were induced in targeted cells by antibody labelling or ingestion of magnetic nanoparticles, RBC and WBC have intrinsic magnetic properties which can be utilized for separation. Deoxygenated RBCs are paramagnetic which means these cells are attracted towards the source of a magnetic field. In contrast, oxygenated RBCs and WBCs are diamagnetic and therefore deflected from magnetic field source [172,173].

Furlani et al. [172] demonstrated isolation of red blood cells and white blood cells from plasma by embedding a microarray of Nickel-based soft magnetic material around the microchannel. When they magnetized the microarray by bringing permanent magnets into proximity, deoxygenated RBCs were attracted and WBCs deflected into two peripheral outlets and blood plasma exited the middle outlet. Using a similar principle, Nam et al. [173] isolated malaria infected RBCs because the malarial parasite produces the by-product hemozoin which makes the sick RBCs develop paramagnetic properties. The achieved a recovery rate of 73% and 98.3% for early and late stage infections, respectively, and a separation efficiency of 99% at 1.6 µL/min flow rate. Robert et al. [174] demonstrated isolation of macrophages and monocytes based on endocytotic properties which have internalized with magnetic nanoparticles. Monocytes are less endocytotic than macrophage and a permanent magnet placed on top of the chamber deflects and sort the cells based on their iron loading. They were successfully sorted with a purity of more than 88% and an efficiency of more than 60% at flow rate of 50 µL/h.

## 8. Multi-Target Separation Utilizing Multiple Cell Properties

As discussed so far, each cell sorting technique has its own advantages and limitations. To separate cells with more nuanced differences, multiple cellular properties should be simultaneously employed by combining techniques in order to improve sorting efficiency.

**Figure 9:**
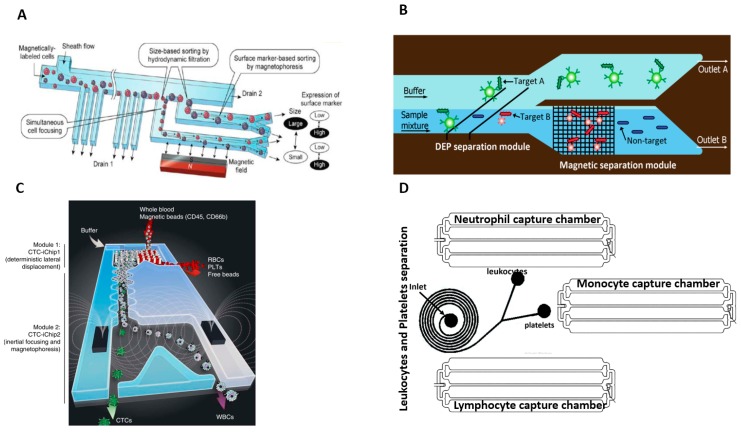
(**A**) Principle of a two-dimensional (2D) cell sorting system integrating hydrodynamic filtration (HDF) and magnetophoresis, Reprinted with permission from Reference [175]. Copyright (2013) American Chemical Society; (**B**) The integrated dielectrophoretic-magnetic activated cell sorter (iDMACS) device architecture with DEP and MACS stage, Reproduced from Reference [176] with permission of The Royal Society of Chemistry; (**C**) Hybrid deterministic lateral displacement (DLD), Inertial and Magnetophoresis device, circulating tumor cells integrated Chip (CTC-iChip) for cancer cell separation, Adapted by permission from Macmillan Publishers Ltd.: [Nat. Protoc.], Reference [110], copyright (2014); (**D**) A schematic of Hybrid Inertial and adhesion based system for leukocytes subtypes.

Mizuno et al. [175] demonstrated a hybrid technique to separate two different cell-types, leukocytes (JM cells) and cancer cells (HeLa cells) and bin all cells from each type by size. In their two stage process, they first used a sheath flow to push cells in a focused flow near the microchannel wall. The stream of cells pass through a series of outlets branching out of the main channel with progressively larger openings, as shown in Figure 9A. Cells exit the microchannel through the first outlet that matches their size. At the end of every outlet, a magnet displaces the magnetically tagged JM cells from the HeLa cells into different outlets to yield groups of leukocytes and cancer cells separated into bins according to size. They achieved a sorting efficiency of 90% and throughput of 10 μL/min.

To separate three different strains of *E. coli* bacteria, Kim et al. [176] combined dielectrophoresis and magnephoresis displacement technique, referred to as integrated dielectrophoretic-magnetic activated cell sorter (iDMACS) as shown in Figure 9B. Bacteria from strain A were tagged with polystyrene beads and deflected by DEP forces to one outlet. Strain B was tagged with streptavidin-coated magnetic particles and captured by a magnet whereas Strain C was unlabeled and allowed to flow through the 2nd outlet. They achieved ~98.9% efficiency for both tagged strains and 100% efficiency for the unlabeled strain at throughput of 2.5 × 10^7^ cells/hour in a single pass separation process.

Karabacak et al. [110] invented a three stage process to separate CTC from whole blood, as shown in Figure 9C. In the first stage, WBCs and CTCs were isolated from other blood cells using deterministic lateral displacement. DLD is a very efficient technique to separate the highly deformable RBCs and small-sized platelets. At the next stage, the collected CTC and WBC cells are lined up by inertial focusing and, subsequently, WBCs (which have been magnetically tagged) are separated from CTCs by magnetophoresis. The WBCs were tagged instead of CTCs because antibody tagging may induce cytotoxicity on the CTCs and impede downstream analysis of the isolated cells. Furthermore, some types of CTCs (of epithelial origin) do not express the binding antibody and thus cannot be labeled. Inertial focusing of WBCs and CTCs prior to magnetophoretic separation improves separation efficiency because all cells flowing in a single focused stream will experience very similar magnetic forces. With this combination of techniques, the high throughput action of inertial and DLD separation could be combined with the precision of magnetophoresis separation. Furthermore, viability of the separated cells, namely CTC, is preserved for accurate downstream analysis. They achieved a 97% yield of rare cells with a processing throughput of 8 mL/h of whole blood.

To separate immature RBCs from the blood of pregnant women, Huang et al. [177] also demonstrated a DLD separation step followed by intrinsic magnetic separation. Immature or nucleated RBCs (NRBCs) are indicators of fetal health and are less deformable than mature RBCs. DLD separates the NRBCs and WBCs from mature RBCs and, at the subsequent stage, a magnetic column deflects the WBCs and attracts the NRBCs into separate outlets. They achieved efficiency of 99% at a throughput of 27 mL/h.

Huang et al. [178] demonstrated a hybrid DEP-immunocapture system to extract prostate cancer cells (LNCaP line) from peripheral blood. They functionalized the device surface to capture antigens expressed by the cancer cells. Through careful tuning of an applied AC electric field, negative DEP forces were generated to repel the non-specific leukocytes while attracting the cancer cells for adhesion through positive DEP forces. This combination of techniques yielded improvements in the capture efficiency up to 85% and purity of <5% leukocytes with a modest throughput of 0.2 mL/h.

Gupta et al. [179] demonstrated separation of multiple cell targets with a single round of separation with increased purity, yield, and throughput. They integrated inertial separation with adhesion sorting in two stages, as shown in Figure 9D, to separate various types of leukocytes from platelets. In the first stage, they used a spiral device to separate leukocytes from platelets. In the second stage, the leukocytes pass through a series of capture channels, each having different leukocyte binding moieties to capture individual leukocyte types like monocytes, neutrophils, and lymphocytes. They achieved purity of 91% and yield of 80% at throughput of 1.5 mL/min.

Hybrid techniques enable more selective and efficient separation of multiple cell targets from a mixture of multiple cell types.

## 9. Challenges and Future Research Directions

Beyond proof of concept demonstrations, the microfluidic methods reviewed are promising methods to implement new clinical applications. Individually, each method has limitations to be overcome and we now briefly discuss the key challenges for future research.

The antibody labelled approach demonstrates good results in purity, recovery rate, and separation. However, the antibodies remain attached to the labelled cells even after isolation, and are difficult to remove from the surface of the cells. Properties and functions of target cells may be altered by the antibodies and this is motivation to develop methods to detach the antibody, especially for applications that require the target cells to be reintroduced into the human body after analysis, such as differentiated stem cells and other healthy cells. In some designs, it is not clear if the limitations can be solved, i.e., where joule heating from magnetic field switching may denature proteins and irrevocably damage or destroy the cells.

Similar drawbacks also limit isolation based on cell surface marker properties. Target cells captured by adhesion to a functionalized surface are difficult to detach. Further optimizations are necessary to increase the low throughput and also to reduce non-specific binding of cells to fully realize the potential for selecting cell targets accurately from a mixture of many cell types in a single pass.

Where cells have distinct intrinsic electrical properties, precise and selective isolation with high recovery rate can be attained with DEP force by tuning the electric field frequency. However, many cell types have similar dielectric properties and this technique cannot be used to efficiently separate them [180]. Methods that separate cells by density and compressibility also permit selection of target cells through tuning of the acoustic wave frequency. Separability becomes an issue when cell types have a broad range or an overlap of size and densities. Both dielectrophoresis and acoustophoresis systems operate with limited flow rate. Beyond a threshold speed, sorting purity degrades because the induced forces are too briefly applied to the target cells.

In contrast, size-based separation with inertial forces requires higher flow rate. Membrane filtration is another common approach to separate cells by size but the membrane is prone to pore clogging by larger cells and this reduces the efficiency of cell sorting and coalescence of pores leads to less capture efficiency as targeted cells may flow through the pores and not be captured. DLD micro-devices do not require dilution of blood sample but these demonstrations either operate at low flow rate or are susceptible to clogging between the micropillars.

All methods reviewed except inertial devices operate at low throughput. Also, all techniques have demonstrated separation between only two different cell types with dilution. In contrast, the commercial parsortix system^®^ demonstrated successful isolation of CTCs from whole blood with almost no dilution [73].

Utilizing several properties like size, shape and deformability simultaneously leads to improved selectivity of target cells from the heterogeneous cell mixture, even when differences between cell types are subtle. Hybrid techniques combining two or more principles for separating cells may be mutually complementary and overcome limitations of individual techniques. In oncology research, microfluidics plays a vital role to isolate rare cancer cells from the normal cells as a preparatory step for further sequential and downstream analysis. High recovery rate with minimal background contamination (high purity) is critically important for downstream analysis to enable clear and reliable investigation of specific cell types. In addition, high throughput is necessary to isolate a sufficient quantity of these rare cells for processing, typically at least 1–10 mL of raw blood while maintaining the cell viability.

## 10. Conclusions

Microfluidic devices have numerous advantages over the traditional bench top methods and are emerging as a new platform for enabling applications in clinical diagnostics and therapeutics such as in oncology. Though the microfluidic device is sterile and disposable, the limitation lies in the capability to process a large sample volume. Though there has been progressive improvement to the methods reviewed, there is often competing tradeoffs between recovery rate, purity, throughput and viability for approaches targeting a single cell property. Hybrid techniques utilizing several cellular properties are a promising approach to isolate multiple cell types by exploiting the benefits of multiple cellular properties in a single pass process.
